# Single-Kernel FT-NIR Spectroscopy for Detecting Supersweet Corn (*Zea mays* L. Saccharata Sturt) Seed Viability with Multivariate Data Analysis

**DOI:** 10.3390/s18041010

**Published:** 2018-03-28

**Authors:** Guangjun Qiu, Enli Lü, Huazhong Lu, Sai Xu, Fanguo Zeng, Qin Shui

**Affiliations:** 1 College of Engineering, South China Agricultural University, Guangzhou 510640, China; qiuq16@scau.edu.cn (G.Q.); vanco5211@sina.com (F.Z.); difafa_shu@126.com (Q.S.); 2 Guangdong Academy of Agricultural Sciences, Guangzhou 510640, China; huazlu@scau.edu.cn; 3 Public Monitoring Center for Agro-Products of Guangdong Academy of Agricultural Sciences, Guangzhou 510640, China; xusai1991@sina.cn

**Keywords:** FT-NIR spectroscopy, supersweet corn, seed quality, nondestructive, single kernel, viability, discriminant analysis

## Abstract

The viability and vigor of crop seeds are crucial indicators for evaluating seed quality, and high-quality seeds can increase agricultural yield. The conventional methods for assessing seed viability are time consuming, destructive, and labor intensive. Therefore, a rapid and nondestructive technique for testing seed viability has great potential benefits for agriculture. In this study, single-kernel Fourier transform near-infrared (FT-NIR) spectroscopy with a wavelength range of 1000–2500 nm was used to distinguish viable and nonviable supersweet corn seeds. Various preprocessing algorithms coupled with partial least squares discriminant analysis (PLS-DA) were implemented to test the performance of classification models. The FT-NIR spectroscopy technique successfully differentiated viable seeds from seeds that were nonviable due to overheating or artificial aging. Correct classification rates for both heat-damaged kernels and artificially aged kernels reached 98.0%. The comprehensive model could also attain an accuracy of 98.7% when combining heat-damaged samples and artificially aged samples into one category. Overall, the FT-NIR technique with multivariate data analysis methods showed great potential capacity in rapidly and nondestructively detecting seed viability in supersweet corn.

## 1. Introduction

Sweet corn (*Zea mays* L. saccharate Sturt) is one of the most popular vegetables in countries such as the United States and Canada and is becoming popular in China and other Asian countries due to its pleasant flavor and high nutritional value [1,2]. This increase in consumer popularity has resulted in an expansion of its planting scale around the world in recent years. However, the low germination rate and seedling vigor of sweet corn seed still limit the development of the sweet corn industry to some extent [3]. Its high soluble sugar content and lower starch content are thought to be the main reasons that sweet corn seeds deteriorate rapidly compared to field corn seeds, especially during the storage process [4,5,6,7]. The reasoning is that less starch means that less endosperm tissue can be reserved as an energy source for metabolism. The process of converting sugar to starch during endosperm development depends on genotype. In particular, supersweet corn seed (with the shrunken-2 allele) can have much higher soluble sugar content (>20%) but lower starch content than sugar-enhanced sweet corn seed (se gene) or any other traditional sweet corn seed (su gene) [8]. As a result, supersweet corn seeds usually exhibit inferior emergence and seedling vigor compared to other sweet corn varieties. Research has indicated that the quality of sweet corn seeds decreases within one year, so new sweet corn seeds, especially supersweet corn seeds, should be used for cultivation every year [9]. In addition, the high soluble sugar content of supersweet corn inhibits the drying of the seed crop in the field. Thus, artificial drying is necessary after harvesting. However, improper temperatures can be harmful to supersweet corn seeds, as drying temperature has a significant effect on germination and storability [10]. For the above reasons, a timely and effective technique for detecting supersweet corn seed quality is of great significance to reduce the risk of inferior and nonviable seeds entering the market and sowing process.

However, the conventional methods of detecting seed viability and vigor, such as the germination test and tetrazolium test (TZ), are time-consuming and destructive to seed samples [11]. The standard germination test, as the official method, provides a germination rate to estimate the capability of a seed lot to produce normal plants with good vigor under favorable conditions, and generally, a period of time is required to follow the operating instructions [12]. The tetrazolium test serves as a faster alternative technique for assessing seed viability, but this test is destructive as well, and its reliance on the standard germination test limits its applicability [13].

Near-infrared (NIR) spectroscopy is commonly used as a fast, nondestructive technique for qualitative and quantitative analyses of organic matter such as agricultural and food products, because the molecular vibrations of the functional groups in organic compounds, which mainly involve stretching and bending vibrations among hydrogen bonds such as C-H, O-H, and N-H, have distinctive overtones; the combination bands have absorption ranges from 780 nm to 2500 nm, namely, the NIR spectrum. In other words, the spectral absorption characteristics in this region are closely related to the organic compounds present. Hence, NIR spectroscopy of organic matter can be utilized to analyze various attributes according to the absorption intensities of specific wavelengths [14]. Since the 1960s, when NIR spectroscopy was successfully utilized by Karl H Norris et al. [15] for the first time to measure the moisture content of seed kernels, many studies have been performed to detect the quality of grain seeds by using the NIR spectroscopy technique at the single-seed level. These studies included nondestructive quantitative detection of chemical characteristics such as moisture, protein, oil, starch, amino acids, and fatty acids in wheat, corn, soybean, rice, and other seeds. Some other applications for qualitative discrimination purposes according to specific physicochemical properties, such as hardness, vitreousness, insect infestation, and mold and toxin infection, have also been studied in various seeds, and detailed descriptions for most of these assays are well summarized in the present literature [16,17,18].

With the improvement of the available instrumentation and analytical techniques in the last decade, some researchers have dedicated themselves to detecting seed viability and vigor by using NIR spectroscopy [18]. Viability detection studies of the seeds of grains [19,20,21,22,23,24], vegetables [11,25,26,27,28,29,30], and fruit [31,32,33] have successfully used NIR spectroscopy or a related technique to discriminate nonviable from viable seeds with high prediction accuracies. To the best of our knowledge, no research has been published to date regarding the use of NIR spectroscopy for supersweet corn seed viability detection.

In this study, it was assumed that deterioration in the viability of supersweet corn seeds was caused by either excessive heating during the drying process or improper storage conditions, which resulted in seed death. Microwave heating and artificial aging experiments were designed to simulate seed viability loss during the above two processes. Fourier transform near-infrared (FT-NIR) spectroscopy was utilized to distinguish the treated supersweet corn seeds from the untreated ones because of its significant advantages, such as high signal-to-noise ratio, high-resolution, and accurate frequency determination by measuring all wavelengths simultaneously. This study mainly aimed to verify the feasibility of using NIR spectroscopy to recognize damaged supersweet corn seeds caused by overheating and accelerated-aging treatments. Furthermore, various preprocessing methods were tested on the embryo-side and endosperm-side spectra when establishing the partial least squares discrimination analysis (PLS-DA) models to determine optimized models for different detection purposes.

## 2. Materials and Methods

### 2.1. Seed Selection and Deterioration Treatment

Three hundred seeds selected from Huameitian No. 8, a favorable supersweet corn variety with the sh2sh2 genotype, were used in this study. Huameitian No. 8, which was bred by the College of Agriculture, South China Agricultural University, contains high soluble sugar content ranging from 21.12 to 22.09% [34,35]. The seed materials were provided by Guangdong South China Agricultural University Seed Industry Company Limited, with an initial seed germination rate of over 95%. After excluding cracked, broken, and discolored seed kernels, 300 seed kernels were selected randomly and divided into three groups evenly (100 per group). The first group was subjected to a deterioration treatment, combining the methods of Wang et al. [36] and Lohumi et al. [31] as follows: the moisture content of the seeds was tempered to 20% in a sealed Erlenmeyer flask for three days before treatment, and then, the seeds were put into a plastic bag and treated by incubation for seven days in a water bath maintained at 45 °C. After treatment, the seeds were dried to their initial moisture content by incubation at 20 °C. The second group was subjected to a microwave treatment by following the method of Agelet et al. [22]. The seed samples were treated three times intermittently for 30 seconds each time, ensuring that no exterior changes could be recognized by the naked eye but achieving a notable effect on seed germination capability. The third group was used as the control group (with no treatments). In addition, all seed materials were placed at an environmental temperature of 25 ± 1 °C and relative humidity of 50 ± 3% for 10 days; the spectrometer was placed in the same conditions before collecting the spectral data to reduce the impact of environmental factors on the spectra.

### 2.2. FT-NIR Spectroscopy Acquisition

**Figure 1:**
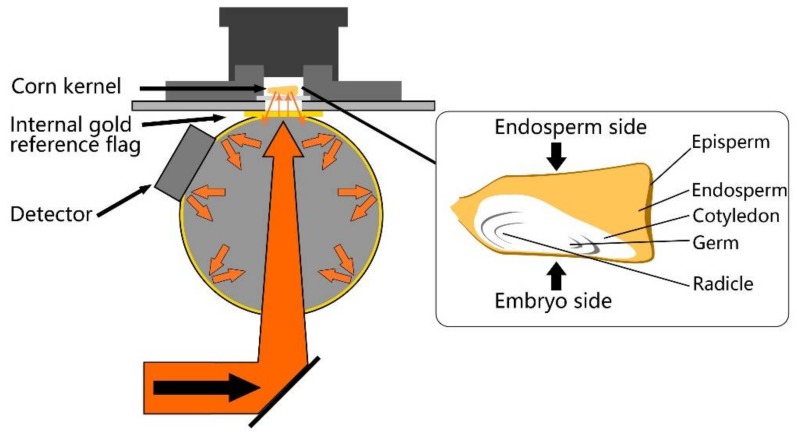
Schematic of Fourier transform near-infrared (FT-NIR) spectroscopy diffuse reflection measurement mode with integrating sphere (**left**) and structure of supersweet corn kernel (**right**).

A diffuse reflection measurement mode was applied in this research to capture the NIR spectroscopy of individual supersweet corn seed kernels (Figure 1). Because the embryo portion strongly correlates to seed viability and the endosperm has the function of storing energy components for germination, which expresses as vigor, both sides of each kernel were scanned. As variations in seed curvature [37], shape [16], roundness, and thickness [24] were found that would add spectroscopic variance, which was irrelevant to any compositional change in the seeds, an integrating sphere was utilized to improve the signal-to-noise ratio, addressing the problem of heterogeneity among the seed kernels.

Three hundred seeds in total were scanned seriatim using an Fourier transform infrared spectrometer (Antaris II FT-NIR Analyzer; Thermo Scientific Co., Waltham, MA, USA). Single supersweet corn seeds were placed in a special sample cup designed for small solid particles. The spectrum of each kernel was scanned in the wavelength range from 10,000 to 4000 cm^−1^ (1000–2500 nm) at 4 cm^−1^ intervals (a 0.6 nm resolution at 1250 nm). The NIR reflectance spectra were expressed in the form of log(1/R), where R is the reflectance. The average spectrum from 32 successive scans of each individual seed was obtained for further analysis. 

### 2.3. Germination Test

The germination test was conducted following the guidelines of the International Seed Testing Association (ISTA) [12] to determine seed viability after spectral data collection. All seed samples were stored in a climatic cabinet at 25 °C. The viability of all seeds was checked daily during the seven-day germination process. Seeds with a 5-mm length germ were counted as germinated (viable) and otherwise as nonviable. The seeds in the control group had a germination rate as high as 98%. However, the germination rates of the seeds that had been subjected to the overheating and artificial aging treatments were quite low, only 2% and 5%, respectively. Although these seeds had the ability to germinate, they were also marked as nonviable due to their weak roots, which could no longer support healthy seedlings.

### 2.4. Dataset and Model Verification

The spectral vectors combined with category variables were imported into the Unscrambler software (Camo AS, Trondheim, Norway) for organization purposes. MATLAB (MathWorks, Natick, MA, USA) with PLS Toolbox v.8.2.1 (Eigenvector Research Inc., Wenatchee, WA, USA) was used to test the various spectral preprocessing and to calculate the correspondence classification models.

**Figure 2:**
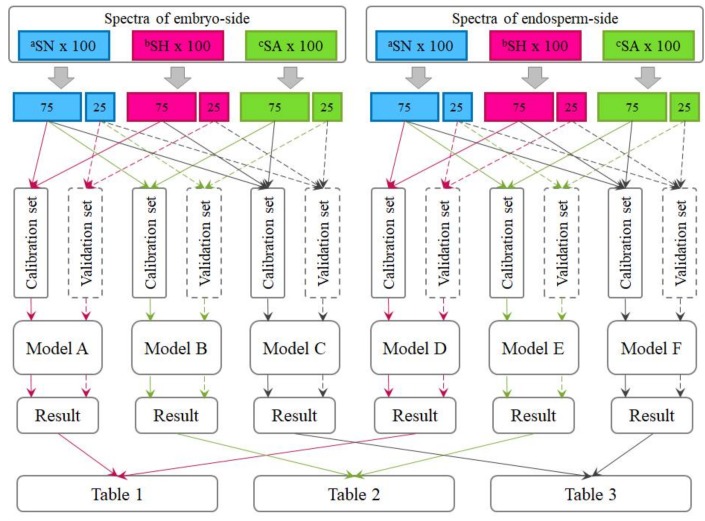
The workflow chart of sample sets: (1) Model A and D for heated-damaged detection; (2) Model B and E for artificially aged detection; and (3) Model C and F for comprehensive discrimination. Note: ^a^SN, spectra of normal samples; ^b^SH, spectra of heated-damaged samples; and ^c^SA, spectra of artificially aged samples.

Six hundred spectra, which were collected from both the embryo side and endosperm side of three hundred kernels, were utilized in this study to establish models for predicting seed viability under each type of treatment. In total, three kinds of models were built as Figure 2 shows, namely, heat-damaged versus normal (Model A and D), artificially aged versus normal (Model B and E), and heat-damaged and artificially aged versus normal (Model C and F).

For each of these six sample types, one-fourth of the samples (one spectrum chosen from every four spectra in the database) was segregated as a validation set, while the remaining three-fourths were utilized as a calibration set for calibrating the discriminant models. In other words, the validation set consisted of samples that were not used to calibrate the models. As a result, when calibrating models for detecting heat-damaged or artificially aged kernels individually, the number of samples in the calibration set was 150 (75 viable and 75 nonviable), and the number of samples in the validation set was 50 (25 viable and 25 nonviable). In addition, when calibrating models to detect the two types of nonviable seeds simultaneously, the heat-damaged samples and the artificial-aged samples merged into one group as nonviable. In this case, the number of samples in the calibration set went up to 225 (75 viable and 150 nonviable), and number of validation samples also went up to 75 (25 viable and 50 nonviable). It should be noted that the spectra collected from different sides of kernels would not appear in a same model, these two-sides spectra were used separately for different analyses purposes.

Two verification methods were applied in this study to verify the performance of the calibration models. On the one hand, 10-fold cross-validation was used to verify whether the model had been fitted or underfitted. Different subsets were determined through random selection of samples in the calibration set. The averaged accuracy results of five iterations were reported for the cross-validation results. On the other hand, Y-scrambling randomization tests were also applied to verify whether there was chance correlation in our models. In the case of the Y-scrambling randomization tests, a random mean distribution generating function was used to randomize the dependent (Y) values. Then, the same preprocessing methods and parameters obtained from the best model were tested on the randomized dataset for calibration and validation purposes. The classification results of 100 iterations were used to evaluate model performance. If the classification accuracy of the new model was much lower than the model based on the original data, it can be concluded that the original model is reasonable [38].

### 2.5. Spectral Data Preprocessing

Spectral data usually contains systemic noise caused by instrument drift and environmental changes that do not contribute to the analysis of the specific property. Correcting the raw spectral data through various preprocessing methods can effectively improve the accuracy and robustness of the model. Smoothing methods are known to reduce the high-frequency part of the noise for each spectrum, and all spectra were subjected to a Savitzky–Golay smoothing treatment (21-point width and third-order polynomial) in advance in this study. Normalization can be used to convert all spectral data to approximately the same scale; a maximum normalization algorithm, which sets the maximum of each row to 1 and divides each variable by its maximum absolute value, was applied in this study. Multiplicative scatter correction (MSC) can handle both the additive and multiplicative effects of light scattering in spectral data, and thus, the offset shifts caused by path-length variations, particle size, or any other similar effects can be successfully treated with MSC. This study used the mean spectrum as a reference when processing MSC, as is common practice. The Savitzky–Golay 1st and 2nd derivatives were applied to correct for baseline effects in the spectra for the purpose of removing nonchemical effects and creating robust calibration models, and a 21-point window and a third-order polynomial were applied for all derivatives preprocessing. Derivatives may also aid in resolving overlapping bands, and thus, they can provide a better understanding of the data, emphasizing small spectral variations not evident in the raw data. The selection of a suitable preprocessing method should always be considered in relation to the successive modeling stage.

### 2.6. Partial Least Squares Discriminant Analysis

PLS-DA, which adds a threshold to classify samples, is a modified version of partial least squares regression (PLSR). This method is commonly used in NIR spectroscopy applications due to its advantages in addressing collinearity problems and overlapping phenomena in NIR spectroscopy analyses. On one hand, it assigns greater weights to the variables that are highly correlated to response variables than to others, and then, the characteristic wavelengths can be easily found within the whole spectral range. On the other hand, redundant information and noise can be removed to a large extent, while creating latent variables that are orthogonal to each other but at the same time most correlated to response variables. The PLS-DA model is explained as follows:

(1) Y= XB+E

where Y is a matrix (a vector when only one response variable is present) that relates to the actual category properties of the samples, X is an n×m matrix that holds the predictor (spectral matrix) values for each sample, B is matrix of regression coefficients for the predictor values, and E is the residual matrix of the portion that has not been explained in X and Y.

To find a linear relationship between the predictors and response variables, both X and Y are decomposed by latent variables (which may be described as factors or components in other publications) such that:

(2) $$X=\;TP^{T}+E_{X}$$

(3) $$Y=\;UQ^{T}+E_{Y}$$

where T and U are the scores matrix and P and Q are the loading matrix. Furthermore, the T scores capture the part of the structure in X that is most predictive for Y, and the U scores summarize the part of the structure in Y that is explained by X along the given factors. $E_{X}$ and $E_{Y}$ are the residual matrices of X and Y, respectively. Indeed, PLS can function with many latent variables. However, using redundant latent variables can lead to model overfitting, because redundant latent variables would introduce noise into the models. In this study, the number of latent variables was chosen when the accumulative variance in Y first achieved 90%. For this study, the spectral data were arranged in X, while the Y matrix (vector) was set up with 1 and 0 as the response variables to indicate the sample categories (i.e., value 1 if sample belongs to the class and 0 if not). In fact, the model could not predict a 1 or 0 value perfectly; hence, a threshold was utilized to determine classification performance. Then, accuracy, which was measured by the number of correctly classified kernels, was used to determine model performance in this study.

## 3. Results and Discussion

### 3.1. Spectral Interpretation

**Figure 3:**
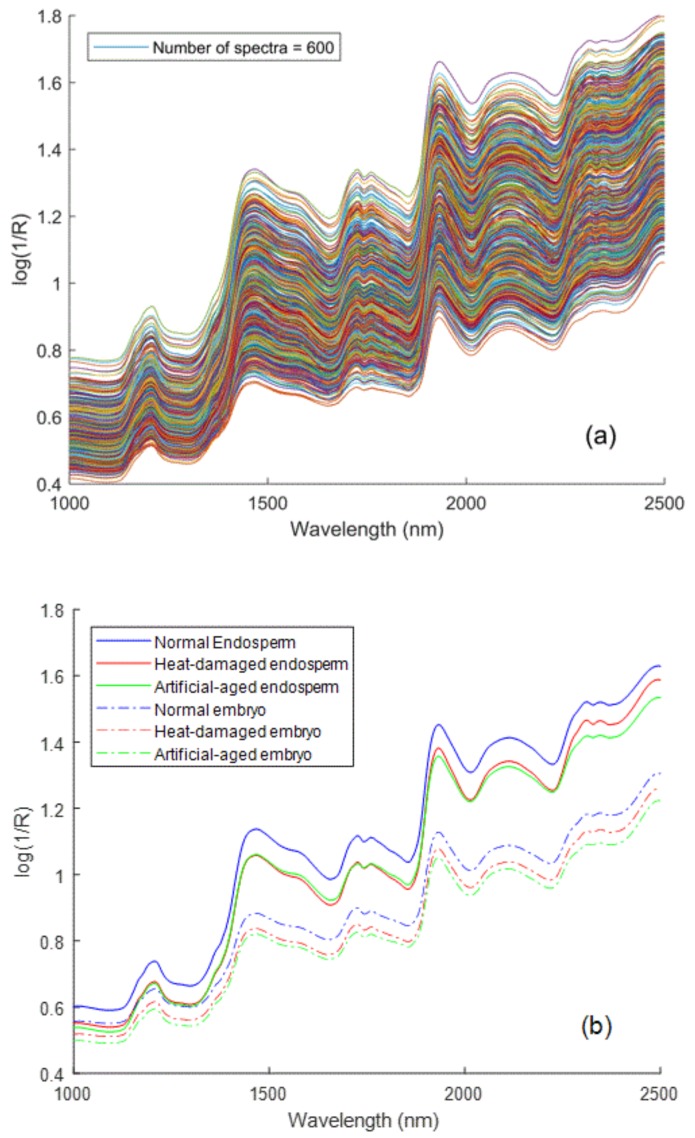
FT-NIR spectra collected from supersweet corn seeds: (**a**) raw spectra and (**b**) mean spectra.

Six hundred sample spectra were utilized, and no outliers were removed because no samples were found outside the Hotelling’s T^2^ ellipse (with confidence level 99%) for the first two principal components plots of each spectra group. Finally, all the raw spectra were plotted in Figure 3a. The wavelength region of 1100–1300 nm in the NIR reflectance spectra shows a single absorption centered at approximately 1200 nm, which is the second overtone of the C-H functional group vibration band [39]. The absorption band at 1470 nm was assigned to the vibrations of the 1st and 2nd overtones of symmetric and asymmetric N-H functional group stretching. Furthermore, the absorption region between 2100 nm and 2350 nm is the combination vibrational band absorption of -NH, -OH, -CH, and similar functional groups. The mean spectra calculated from the raw spectra of the normal, heat-damaged, and artificially aged samples were plotted in Figure 3b. The nonviable seed samples from both deterioration treatments showed lower absorption than that of the viable seeds, consistent with the results of Agelet et al. [20] and Ambrose et al. [22] for microwave heating and Wang et al. [36] and Kusumaningrum et al. [24] for constant-temperature aging. This could be because the microstructure of seed tissue had changed when deterioration treatments were processed, which caused the NIR sensor to detect stronger reflection signals reflected by seed kernels [36]. In other words, differences in light scattering were observed between normal seeds and damaged seeds, and large gaps between the spectra of the normal and damaged samples were found in the regions 1470–1650 nm, 1800–1900 nm, and 2050–2300 nm; this pattern emerged in the spectra from both sides of the seed kernels. In addition, the spectra from the endosperm side had higher overall absorptions than those from the embryo side for all samples in this experiment.

**Figure 4:**
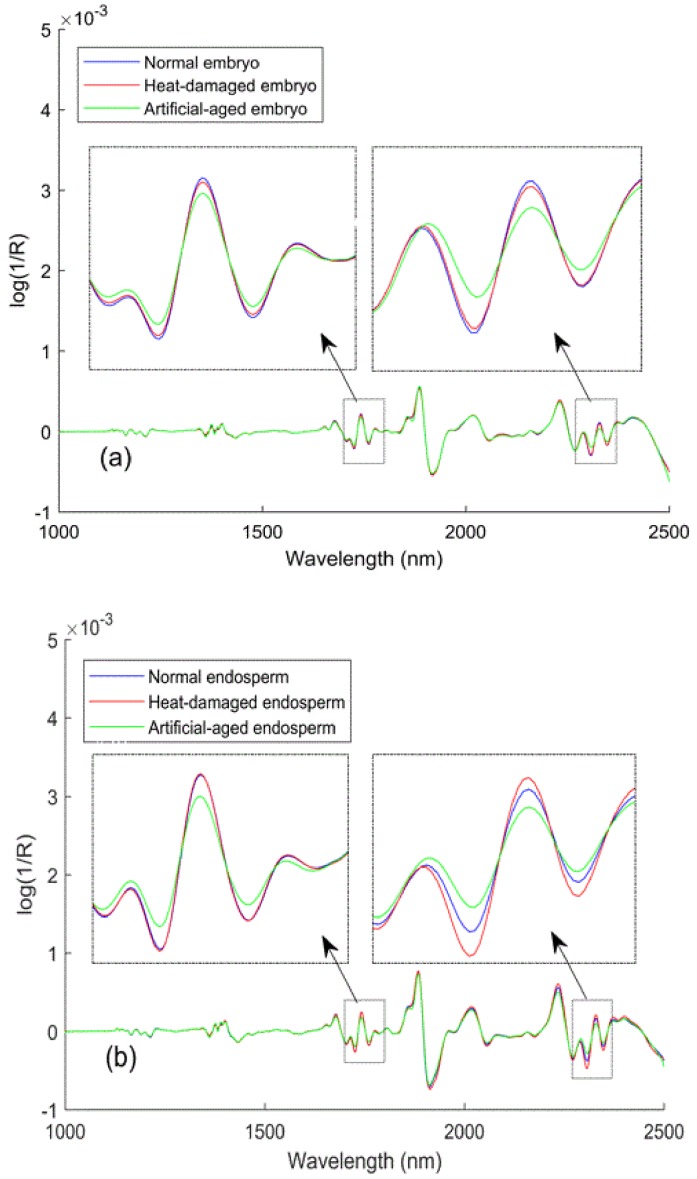
Savitzky–Golay 2nd derivative spectra from mean spectra: (**a**) embryo and (**b**) endosperm.

The spectra after preprocessing with the Savitzky–Golay 2nd derivatives (Figure 4) showed that major absorbance differences between viable and nonviable kernels could be observed both in the first overtone region (1600–1800 nm) and the combination bands region (2200–2400 nm). Differences in the peaks at approximately 1680 nm, 1730 nm, and 1740 nm could be explained as the characteristics of the first overtones of the vibrations from the -CH_3_, -CH_2_, and -CH functional groups, respectively, which related to carbohydrates. Hourant et al. [40] ascribed the absorption at 1710 nm and 1750 nm to correlation with the fatty acids in seeds (i.e., linoleic and oleic acids), which are characteristic components of seeds that are nonviable due to aging. Furthermore, differences from the -CH_3_, -CH_2_, and -CH combination bands were also found at wavelengths of 2310 nm, 2330 nm, and 2350 nm, respectively, which related to carbohydrates as well. This result could be because carbohydrate compounds, including sugar, starch, and cellulose, represent close to two-thirds of the supersweet corn kernel by weight, and thus, changes in these compounds may be more easily detectable. Furthermore, the local, enlarged views show additional information about the variation tendencies that the three sample types showed in response to spectral signals as the wavelengths increased. The lower absolute value of artificial-aged seeds showed that artificial-aged treatment reduced the gradient of the variation tendency in these regions. By contrast, the response pattern of the heat-damaged kernels was more similar to normal kernels. But with an exception, the spectra (2250–2350 nm) collected from the endosperm-side showed that the change rate increased after overheating. From this, it can be assumed that the two kinds of deterioration treatments would not always cause the same change in the supersweet corn seeds, although they all resulted in lower absorption than that of the viable seeds.

### 3.2. Heat-Damaged Kernel Detection Models

The prediction results of overheating-damage detection are summarized in Table 1. Various preprocessing methods were implemented to investigate how the accuracies of the classification models were affected by different preprocessing algorithms and spectral datasets. All PLS-DA models yielded satisfactory accuracy (greater than 90%), which indicated that a difference was present between the normal and heat-damaged kernels. The models discriminated samples with varying accuracies under various preprocessing methods, but the differences among them were not significant. The high classification rate of the raw spectra model implied that light scattering characteristics could be utilized for heat-damaged kernels, which agrees with the results of Wang et al. [36] in wheat heat-damage detection. Savitzky–Golay derivative preprocessing did not exhibit better performance than did other methods, but it reduced the number of latent variables used to reach equivalent accumulative variance in calibrating the models. Compared with those using the spectra of the embryo side, the models established that using the spectra of the endosperm side could generate equivalent accuracies. Hence, although the embryo is the seed part most relevant to viability, the damage characteristics on the endosperm side can also be included as useful features for seed quality evaluation.

The cross-validation of these models resulted in an average 97.5%, with a range from 95.3% to 99.3% (data not shown in Table 1). It indicated that the overfitting phenomenon did not appear in these models. The Y-scrambling test was applied on spectra of the embryo side with MSC preprocessing, because the model generated the highest accuracy for both the calibration set and the validation set based on such combination. The results showed that the average accuracy was 58.4% and 49.2% for the calibration set and the validation set, respectively. And the highest accuracy among the 100 iterations of both datasets was 65.3% and 66.0% for the calibration set and the validation set, respectively. The classification accuracies were obviously lower than the results of the original models. Therefore, we believe that there was no chance correlation in our models, and the present analysis results are feasible.

### 3.3. Artificially Aged Kernel Detection Models

Table 2 summarizes the prediction results of the artificial aging detection models. The normal and artificially aged corn seeds were well-distinguished in all preprocessing models using spectra collected from both sides of the kernels, resulting in high accuracies in the validation sets, from 90.0% to 98.0%. The Savitzky–Golay 1st derivative preprocessing exhibited good performance, with accuracies of 100% and 98.0% for the calibration set and the validation set, respectively, when using the spectra from the embryo side. The decreased performance after preprocessing with the Savitzky–Golay 2nd derivative for the spectra from the endosperm side may be due to the probability that more noise was included in the cumulative variance when calibrating the model. Furthermore, the artificially aged kernel detection results of the models from the embryo-side spectra were overall better than those from the endosperm-side spectra, indicating that the changes caused by artificial aging in the embryonic part were more significant than those in the endosperm part. However, slight changes in accuracy may also be attributed to the heterogeneity caused by the curvature, shape, color, and size of each seed sample [16,37].

For these models, the average accuracy of cross-validation was 98.1%, with the accuracy range between 97.3% and 100%. The Y-scrambling test on the spectral data with Savitzky–Golay 1st derivative preprocessing, which produced the best results in the original model, showed that the average accuracy was 59.2% and 50.2% for the calibration set and the validation set, respectively. At the same time, all accuracy results of these tests were no more than 66%. Based on these results, we made our judgment that there was no overfitting or chance correlation in our models for artificially aged kernels detection.

### 3.4. Comprehensive Discriminant Models

**Figure 5:**
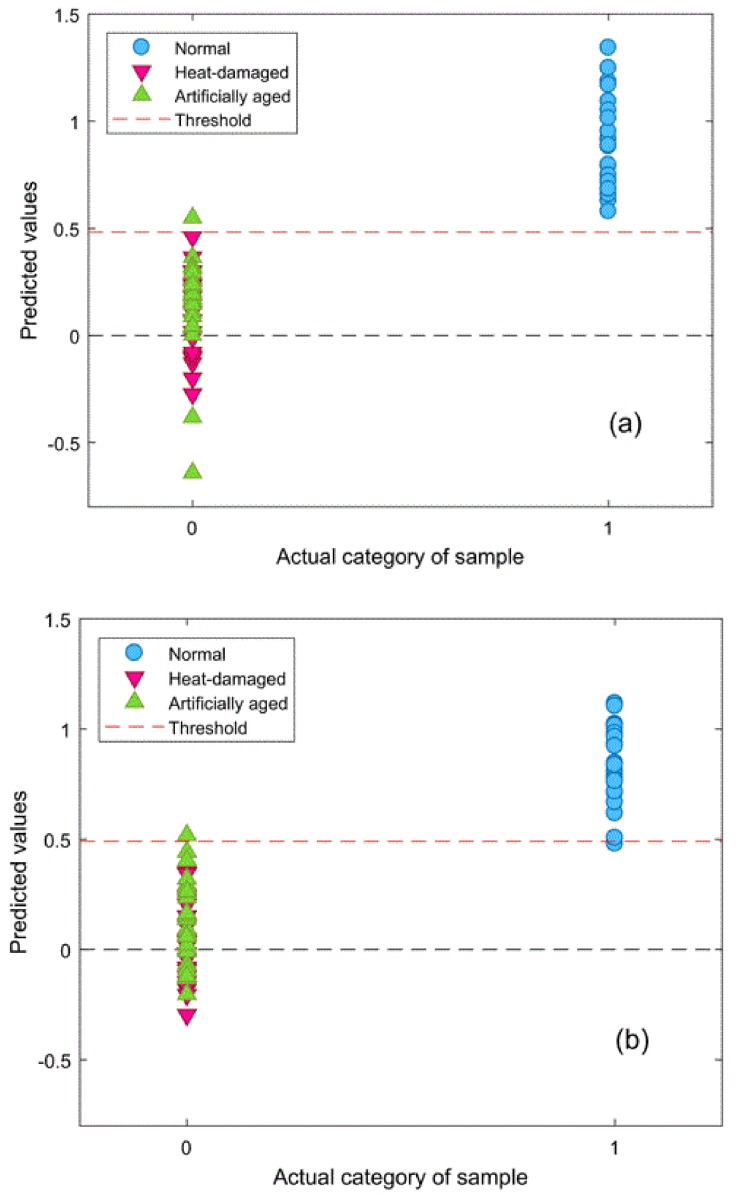
Classification results of the validation set with Savitzky–Golay 1st derivative preprocessing: (**a**) embryo and (**b**) endosperm.

**Figure 6:**
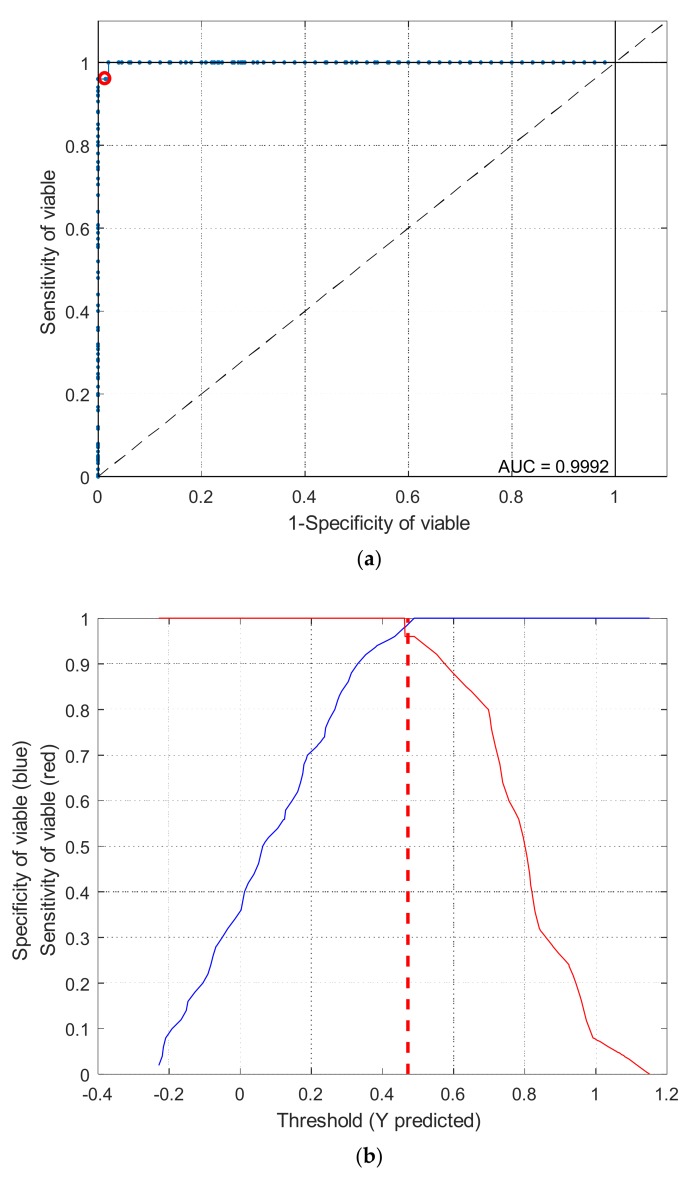
Receiver operating characteristic curves (**a**) and threshold plots (**b**) for comprehensive PLS-DA model with FT-NIR spectral data.

**Figure 7:**
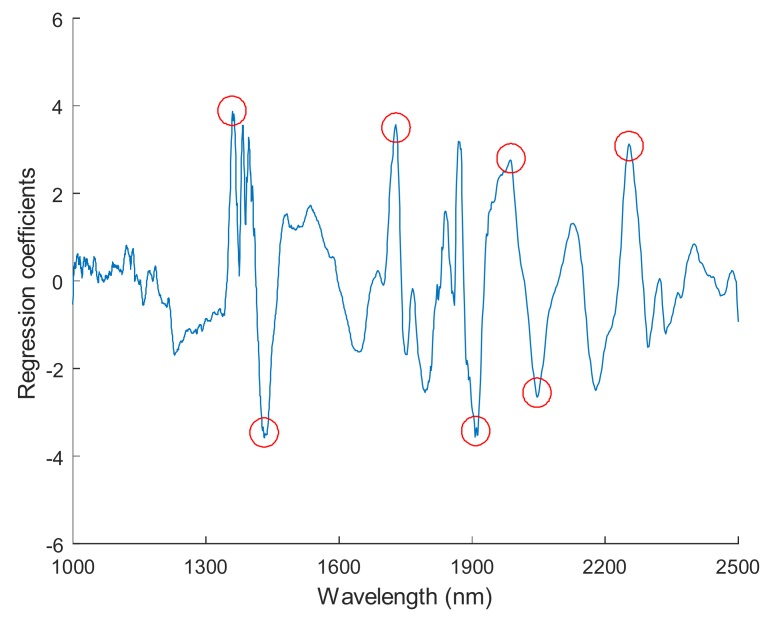
Regression coefficients derived from the comprehensive PLS-DA models with FT-NIR spectral data.

To distinguish viable seeds from both types of damaged seeds simultaneously, comprehensive discriminant models were calibrated by combining heat-damaged samples with artificially aged samples as one category, the nonviable category. The results of the comprehensive discriminant models are described in Table 3. The performances of these models for prediction testing were slightly lower than those of the models built to detect a single type of damaged seeds, with a range of accuracy between 90.7% and 98.7%. Savitzky–Golay 1st derivative preprocessing led to slightly higher accuracies than did the other methods. The classification details plotted in Figure 5 show that damaged kernels could be correctly recognized when modeled with Savitzky–Golay 1st derivative preprocessing. In addition, the appropriate threshold can be attained by the PLS-DA algorithm with a good sensitivity without having to lose too much specificity (Figure 6). Overall, the accuracy of the validation set while modeling with the endosperm-side spectra was higher than that with the embryo-side spectra, except when the data was preprocessed with the Savitzky–Golay 2nd derivative.The regression coefficient calculated by the PLS model, which represents the spectral characteristics among different seed categories, can be used to interpret the results (Figure 7). The peaks and valleys can be assigned to the chemical components changed during the damage treatments. The regression coefficient derived from the comprehensive PLS-DA models showed that the high absolute values of the wavelengths and spectral regions (e.g., 1730 nm, 1910 nm, and 2200–2300 nm) agreed with those differences in the mean spectra plots (Figure 3b) and their Savitzky–Golay 2nd derivative plots (Figure 4). 

In addition, the peaks at approximately 1359 nm may be related to C-H 1st overtone vibration absorption, and the observed valley at approximately 1400–1450 nm corresponds to the 1st overtone of O-H stretching [21] and may also be related to the C-H 2nd overtone stretching due to absorption by the CH_3_ functional group [22]. The peak at 1728 nm is related to the C-H 1st overtone stretching due to absorption by CH_2_ and CH. Several characteristic points were observed in the higher wavelength region (1900–2300 nm), which notably represented compounds such as carbohydrates and proteins, the two most prevalent fractions in supersweet corn seed kernels. For instance, the high absolute values at 1913 nm and 1987 nm, which correspond to C=O stretching, and 2254 nm, which corresponds to O-H stretching, may represent carbohydrate content, and at 2046 nm, which corresponds to N-H stretching vibration overtones, represents protein content [41]. To a certain extent, these observations verified previous results showing that accelerated aging changed the contents of carbohydrates, reducing sugars, proteins, and amino acids in corn seeds [42]. However, Ambrose et al. [22] reported that differences in sample materials and experiment instrumentation would cause variation in the peaks and valleys between different studies, and some minor differences from previous studies on corn kernels were also found in this study [20,21,22,23].

**Figure 8:**
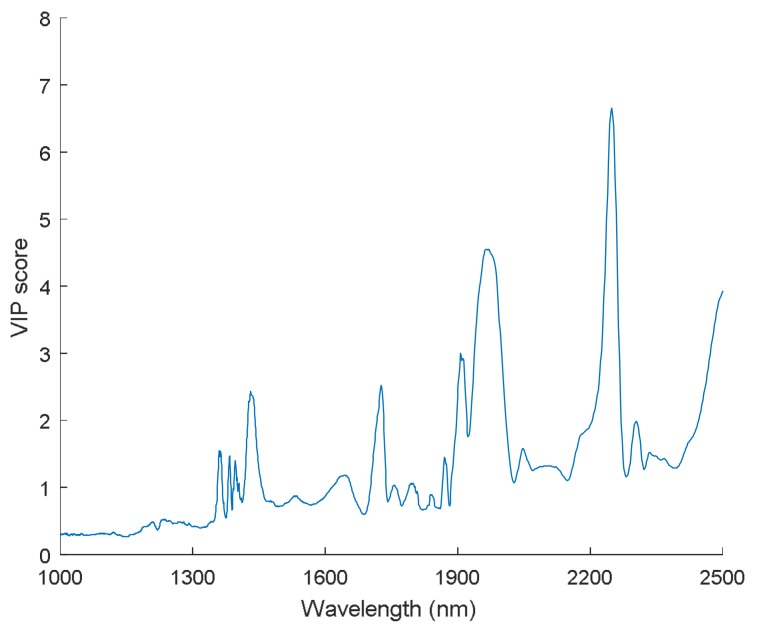
Variable importance in projection (VIP) calculated from the comprehensive PLS-DA models with FT-NIR spectral data.

The variable importance in projection (VIP) can help to inspect the contribution of each variable in spectra that describes the relationship between the spectral matrix (X) and response vector (Y) [43]. Five evident peaks (with a score greater than 2) could be observed in the VIP score plot (Figure 8), which confirmed some feature wavelengths again that we found in Figure 7, such as 1728 nm, 1910 nm, and 2254 nm. Hence, the VIP score can also provide information for analyzing changes of chemical properties in samples.

## 4. Conclusions and Outlook

The appropriate accuracies of the prediction models demonstrated that FT-NIR spectroscopy with multivariate data analysis could be used to detect nonviable supersweet corn seeds that have been damaged by overheating and artificial aging, indicating that the NIR technique has great potential value in inspecting seed quality at the single-kernel level. Spectra collected from the embryo sides and endosperm sides of corn kernels had almost the same capacity to predict viability. This result may be reasonably explained by the presence of global physical and chemical changes caused by the deterioration process, which were captured by NIR spectroscopy, especially the properties of chemical compounds such as carbohydrates and proteins that play an important role in seed germination. Thus, NIR spectroscopy could be applied as a rapid, nondestructive technique to develop a sorting system for supersweet corn seeds.

However, the reasons that seeds cannot germinate even under suitable conditions are diverse and complex; thus, more studies on detecting seed nonviability caused by other reasons such as frost damage during the growth process and natural aging could be performed, although no feasible result using NIR spectroscopy has yet been published [20]. This lack may be due to the effects of seed heterogeneity and the detection limitations of the NIR technique. Hence, more scientific collection schemes and other nondestructive techniques such as mid-infrared (MIR) spectroscopy, Raman spectroscopy, and hyperspectral imaging systems could be attempted to evaluate supersweet corn seed quality, because of their advantages in detecting trace substances and working with small samples. Finally, future studies should be conducted with more samples and more varieties in order to calibrate more robust and applicative models for quality detection, and seed samples selected from different batches should also be considered to improve model performance.

## Figures and Tables

**Table 1:** Heat-damaged kernel detection results of the partial least squares discriminant analysis (PLS-DA) models using embryo and endosperm FT-NIR spectra.

		^a^ LVs	^b^ PC			^c^ PV		
			Viable	Nonviable	Total	Viable	Nonviable	Total
Embryo	Raw	10	75/75	75/75	100%	24/25	24/25	96.0%
	Normalization	10	75/75	75/75	100%	24/25	24/25	96.0%
	MSC (mean)	9	75/75	75/75	100%	25/25	24/25	98.0%
	S-G 1st	5	72/75	75/75	98.0%	24/25	22/25	92.0%
	S-G 2nd	6	75/75	75/75	100%	25/25	23/25	96.0%
Endosperm	Raw	10	75/75	73/75	98.7%	24/25	25/25	98.0%
	Normalization	10	75/75	74/75	99.3%	24/25	25/25	98.0%
	MSC (mean)	9	75/75	74/75	99.3%	24/25	25/25	98.0%
	S-G 1st	4	74/75	75/75	99.3%	25/25	24/25	98.0%
	S-G 2nd	4	75/75	74/75	99.3%	24/25	24/25	96.0%

Notes: ^a^ LVs, number of latent variables; ^b^ PC, performance of calibration; and ^c^ PV, performance of validation.

**Table 2:** Artificially aged kernel detection results of the PLS-DA models using embryo and endosperm FT-NIR spectra.

		LVs	PC			PV		
			Viable	Nonviable	Total	Viable	Nonviable	Total
Embryo	Raw	7	73/75	73/75	97.3%	24/25	24/25	96.0%
	Normalization	7	74/75	73/75	98.0%	24/25	24/25	96.0%
	MSC (mean)	6	75/75	74/75	99.3%	24/25	24/25	96.0%
	S-G 1st	4	75/75	75/75	100%	25/25	24/25	98.0%
	S-G 2nd	3	74/75	73/75	98.0%	25/25	24/25	98.0%
Endosperm	Raw	8	74/75	74/75	98.7%	25/25	22/25	94.0%
	Normalization	8	74/75	73/75	98.0%	25/25	22/25	94.0%
	MSC (mean)	7	74/75	73/75	98.0%	24/25	22/25	92.0%
	S-G 1st	6	74/75	75/75	99.3%	25/25	22/25	94.0%
	S-G 2nd	5	75/75	74/75	99.3%	24/25	21/25	90.0%

**Table 3:** PLS-DA models combining both types of nonviable corn seeds.

		LVs	PC			PV		
			Viable	Nonviable	Total	Viable	Nonviable	Total
Embryo	Raw	18	73/75	150/150	99.1%	23/25	47/50	93.3%
	Normalization	18	72/75	150/150	98.7%	23/25	47/50	93.3%
	MSC (mean)	17	72/75	150/150	98.7%	23/25	47/50	93.3%
	S-G 1st	11	75/75	149/150	99.6%	25/25	49/50	98.7%
	S-G 2nd	9	74/75	147/150	98.2%	24/25	47/50	94.7%
Endosperm	Raw	13	74/75	147/150	98.2%	24/25	49/50	97.3%
	Normalization	13	74/75	146/150	97.8%	24/25	49/50	97.3%
	MSC (mean)	12	74/75	147/150	98.2%	24/25	48/50	96.0%
	S-G 1st	9	74/75	148/150	98.7%	25/25	49/50	98.7%
	S-G 2nd	9	75/75	148/150	99.1%	22/25	46/50	90.7%
